# First national assessment of wildlife mortality in Ecuador: An effort from citizens and academia to collect roadkill data at country scale

**DOI:** 10.1002/ece3.9916

**Published:** 2023-03-26

**Authors:** Pablo Medrano‐Vizcaíno, David Brito‐Zapata, Adriana Rueda‐Vera, Pablo Jarrín‐V, José‐María García‐Carrasco, Diana Medina, Juan Aguilar, Néstor Acosta‐Buenaño, Manuela González‐Suárez

**Affiliations:** ^1^ Ecology and Evolutionary Biology, School of Biological Sciences University of Reading Reading UK; ^2^ Red Ecuatoriana Para el Monitoreo de Fauna Atropellada‐REMFA Quito Ecuador; ^3^ Universidad San Francisco de Quito USFQ, Instituto iBIOTROP, Museo de Zoología & Laboratorio de Zoología Terrestre Quito Ecuador; ^4^ Instituto de Investigación en Biomedicina de la Universidad Central del Ecuador Quito Ecuador; ^5^ Dirección de Innovación Instituto Nacional de Biodiversidad Quito Ecuador; ^6^ Department of Animal Biology, Faculty of Science University of Málaga Málaga Spain; ^7^ Parque Nacional Cayambe Coca Zona baja‐Ministerio del Ambiente, Agua, y Transición Ecológica del Ecuador El Chaco Ecuador; ^8^ Escuela de Biología Universidad del Azuay Cuenca Ecuador; ^9^ Ministerio del Ambiente, Agua, y Transición Ecológica del Ecuador Quito Ecuador

**Keywords:** biodiversity hotspot, citizen science, Galapagos, road ecology, threatened species, vertebrates

## Abstract

Ecuador has both high richness and high endemism, which are increasingly threatened by anthropic pressures, including roads. Research evaluating the effects of roads remains scarce, making it difficult to develop mitigation plans. Here, we present the first national assessment of wildlife mortality on roads that allow us to (1) estimate roadkill rates per species, (2) identify affected species and areas, and (3) reveal knowledge gaps. We bring together data from systematic surveys and citizen science efforts to present a dataset with 5010 wildlife roadkill records from 392 species, and we also provide 333 standardized corrected roadkill rates calculated on 242 species. Systematic surveys were reported by ten studies from five Ecuadorian provinces, revealing 242 species with corrected roadkill rates ranging from 0.03 to 171.72 ind./km/year. The highest rates were for the yellow warbler *Setophaga petechia* in Galapagos (171.72 ind./km/year), the cane toad *Rhinella marina* in Manabi (110.70 ind./km/year), and the Galapagos lava lizard *Microlophus albemarlensis* (47.17 ind./km/year). Citizen science and other nonsystematic monitoring provided 1705 roadkill records representing all 24 provinces in Ecuador and 262 identified species*.* The common opossum *Didelphis marsupialis,* the Andean white‐eared opossum *Didelphis pernigra*, and the yellow warbler *Setophaga petechia* were more commonly reported (250, 104, and 81 individuals, respectively). Across all sources, we found 15 species listed as “Threatened” and six as “Data Deficient” by the IUCN. We recommend stronger research efforts in areas where the mortality of endemic or threatened species could be critical for populations, such as in Galapagos. This first country‐wide assessment of wildlife mortality on Ecuadorian roads represents contributions from academia, members of the public, and government, underlining the value of wider engagement and collaboration. We hope these findings and the compiled dataset will guide sensible driving and sustainable planning of infrastructure in Ecuador and, ultimately, contribute to reduce wildlife mortality on roads.

## Graphical abstract

The first national assessment of wildlife mortality caused by road traffic in Ecuador. A comprehensive roadkill dataset with more than 5000 roadkill records in Ecuador that includes threatened and poorly known species. A joint effort of citizens and academia to collect roadkill data at a country scale.

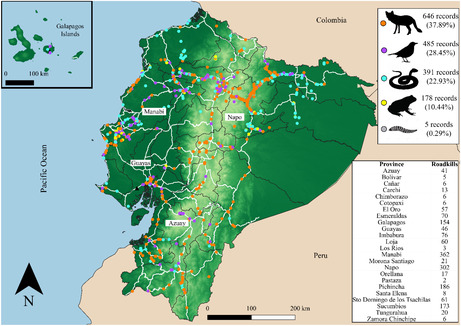

## Resumen

Ecuador tiene gran riqueza y alto endemismo de especies, mismas que están amenazadas por presiones antrópicas como las carreteras. Sin embargo, la investigación en este campo es escasa, dificultando el desarrollo de planes de mitigación. Presentamos la primera evaluación nacional de mortalidad de fauna silvestre en carreteras que nos permite 1) estimar tasas de atropellamiento por especies, 2) identificar especies y áreas afectadas, y 3) revelar vacíos de información. Compilamos datos de estudios sistemáticos y esfuerzos de ciencia ciudadana en Ecuador para presentar una base de datos que comprende 5010 registros de atropellamientos correspondientes a 392 especies, también proveemos 333 tasas de atropellamiento estandarizadas para 242 especies. Monitoreos sistemáticos fueron reportados por diez estudios de cinco provincias del Ecuador, revelando 242 especies con una tasa de mortalidad que varía entre 0.03 y 171.72 ind./km/año. Las tasas de atropellamiento más altas correspondieron a la reinita amarilla *Setophaga petechia* en Galápagos (171.72 ind./km/año), el sapo de la caña *Rhinella marina* in Manabí (110.70 ind./km/año), y la lagartija de lava de Galápagos *Microlophus albemarlensis* (47.17 ind./km/año). La ciencia ciudadana y monitoreos no sistemáticos proporcionaron 1705 registros representando a las 24 provincias de Ecuador y a 262 especies identificadas*.* La zarigüeya común *Didelphis marsupialis*, la zarigüeya orejiblanca andina *Didelphis pernigra* y la reinita amarilla *Setophaga petechia* fueron las más reportadas (250, 104 y 81 individuos respectivamente). Considerando todas las fuentes de datos encontramos 15 especies clasificadas como amenazadas y seis como datos insuficientes por la UICN. Recomendamos mayores esfuerzos de investigación en áreas donde la mortalidad de especies endémicas o amenazadas puede ser crítica para las poblaciones, como en Galápagos. Esta primera evaluación de mortalidad silvestre en carreteras ecuatorianas representa contribuciones de varios sectores, incluidos la academia, ciudadanía y el gobierno, resaltando el valor de una mayor participación y colaboración. Esperamos que estos hallazgos y la base de datos guíen la planificación sostenible de infraestructuras viales en Ecuador y contribuyan a reducir la mortalidad animal en las carreteras por medio de una conducción vehicular más cautelosa.

## 1 INTRODUCTION

Ecuador is a small (283,561 km^2^) but highly biodiverse country hosting two biodiversity hotspots: Choco/Darien/Western Ecuador and Tropical Andes (considered to have the highest richness and endemism of vertebrates species of the planet; Myers et al., 2000). Approximately 659 amphibian species (Ron et al., 2021), 500 reptile species (Torres‐Carvajal et al., 2022), 1699 bird species (Freile & Poveda, 2019), and 466 mammal species (Brito et al., 2021) have been described in Ecuador. Likely, these numbers are highly underestimated as new species are still regularly identified (e.g., Brito et al., 2022; Guayasamin et al., 2022; Leonan et al., 2022) and cryptic species are probably common (Funk et al., 2012). Ecuador is also a highly anthropized country transected by more than 16,647 km of primary and secondary roads (Meijer et al., 2018). A recent study estimated that 420,861 birds and 119,599 mammals are roadkilled in Ecuador each year (Medrano‐Vizcaíno, Grilo, et al., 2022). The problem is compounded with a further 1555 km of new roads planned by 2030 (MTOP, 2016). With this additional road area, roadkill numbers are predicted to increase by 9.3% (Medrano‐Vizcaíno, Grilo, et al., 2022). High road mortality, high biodiversity, and limited information have contributed to propose this territory as a priority for road ecology research (Medrano‐Vizcaíno et al., 2023). Indeed, systematic research, published as indexed publications and theses, focused on wildlife mortality on roads is scarce in Ecuador. To our knowledge, the first study of this type was conducted in 2007, in the Galapagos Islands but focused on a single species, the Galapagos lava lizard (*Microlophus albemarlensis*) (Tanner & Perry, 2007). The first study in continental Ecuador was conducted in 2014 and reported broadly on all tetrapods detected (Medrano‐Vizcaíno, 2015). Although a few additional studies have been published since then (Aguilar et al., 2019; Armendáriz, 2022; Filius et al., 2020; Gaón & Valdez, 2021; García‐Carrasco et al., 2020; González, 2018; Zavala, 2020), research is still limited. This paucity of road impact assessments is common to all Latin America (Pinto et al., 2020), and poses a great challenge to assess potential threats to biodiversity.

Understanding the effects of roads on wildlife populations is key for conservation plans that include risk assessment, planning of future roads, and mitigation of current impacts. However, conducting systematic roadkill surveys is costly and requires funding for fieldwork, which can be scarce or unavailable in developing countries. New technology and internet access have provided the opportunity for new ways to gather data with citizens being involved in science projects as active collaborators. Citizen science is a valuable approach that engages diverse people, and offers a way to obtain field data without high costs (e.g., Medrano‐Vizcaíno et al., 2020; Mueller et al., 2019). Data on road impacts obtained from citizen science projects can complement systematic surveys. Indeed, several citizen science projects have collected roadkill data and contributed to inform about the magnitude of road impacts on wildlife in different parts of the world (see Chyn et al., 2019; Périquet et al., 2018; Raymond et al., 2021; Swinnen et al., 2022; Valerio et al., 2021). Moreover, involving the public with the collection of roadkill data offers an opportunity to provide environmental education and awareness in local communities (Vercayie & Herremans, 2015), which could significantly contribute to reduce wildlife mortality on roads.

The scarce and disperse information on the ecological impact of roads in Ecuador has limited the potential to identify the areas and species most affected. Lack of information has likely prevented the development of environmental policies and concern of road impacts during the planning of new infrastructures. As a first step to address the knowledge gap, we have consolidated a national database consisting of >5000 roadkill records from systematic studies and nonsystematic observations that represent 392 identified wild species. Analyzing this database, we calculated standardized roadkill rates for data coming from systematic surveys, identified species with high mortality that may require urgent protection measures, and revealed unstudied areas (research gaps) where citizen and scientific efforts are needed to best understand the impacts of roads on Ecuadorian wildlife. Compiling this data required collaboration among citizens and academics. We hope to raise awareness of the issue of road impacts on wildlife in Ecuador and encourage policymakers and researchers to work together to collect needed data to guide conservation plans and sustainable roads.

## 2 METHODS

Data from systematic studies often control and report sampling efforts, thus allowing us to estimate standardized roadkill rates (i.e., number of individuals killed per area and time span) for different species and areas. However, these studies, and thus their data, are often limited to particular road sections and periods of time. On the other hand, citizen science data (nonsystematic surveys) can cover wider spatial and temporal windows, but sampling is often not systematized or quantified, which makes the definition of standardized estimates complicated. To avoid methodological issues, data obtained from these two approaches are presented separately.

### 2.1 Records from systematic studies

We completed a comprehensive search for roadkill surveys conducted in Ecuador, considering only peer‐reviewed publications and BSc, MSc, or PhD theses that reported data on the length of road surveyed, taxonomic information of roadkilled species, number of roadkills per species, and survey period. The bibliographic search was performed between March and May 2022 using Google Scholar, Scopus, Web of Science, and PubMed Advanced Search Builder (ASB) with keywords in English and Spanish. The use of both languages was necessary given that Ecuador is a Spanish‐speaking country, and some studies may be published in that language. No time limit was set for the search, if they met the required eligibility criteria, all publications irrespective of date were included. We used the following search strings: Search 1: “Ecuador*” AND (“roadkill*” OR “wildlife mortality” OR “wildlife‐vehicle collision*” OR “wildlife vehicle collision*”) AND (“animal*” OR “amphibian*” OR “bird*” OR “avian*” OR “mammal*” OR “reptile*” OR “snake*” OR “frog*”). Search 2: “Ecuador*” AND (“atropellamiento*” OR “colisión” OR “colision*”) AND (“animal*” OR “fauna” OR “vida silvestre” OR “silvestre*” OR “anfibio*” OR “ave*” OR “pájaro*” OR “mamífero*” OR “reptil*” OR “serpiente*” OR “culebra*” OR “sapo*” OR “rana*”). The authors were contacted for those publications that did not disclose the original dataset; otherwise, the published dataset was downloaded and processed. From each found dataset, we collected data on the following variables: taxonomic identification of roadkilled organisms, number of roadkills per species or lowest identified taxonomic group, length of road surveyed, survey method (e.g., car, motorcycle, bicycle, or walking), time interval between surveys, date of survey, total sampling period, and geographical coordinates of each roadkill record when available. As original records could include synonyms or obsolete species names, we standardized taxonomic names with the IUCN nomenclature. We removed any records of domestic and farm animals. For each dataset, we calculated roadkill rates per species (only specimens identified to species were considered) by dividing the total number of records by the total length in kilometers of the surveyed road(s) and the total sampling period (i.e., number of days between the first day until the last day of fieldwork). The obtained values were transformed into standardized roadkill rates as the number of individuals per km per year multiplying by 365. Additionally, as detectability and survey intervals can influence the number of individuals detected as roadkill, we applied some correction factors. Roadkill rates were multiplied by correction factors based on taxonomic groups, body mass, and survey intervals, proposed by Santos et al. (2011). Additionally, as surveying roads by driving a car could result in a lower carcass detectability, roadkill rates were also multiplied by correction factors proposed by Wang et al. (2022). A full list of these correction factors is provided in Supporting Information Appendix S1. Standardized corrected roadkill rates allow for better comparison of road mortality among species and areas where systematic studies have been conducted.

### 2.2 Citizen science and other records (nonsystematic monitoring)

Our main data source was our citizen science project, entitled “Red Ecuatoriana para el Monitoreo de Fauna Atropellada” (REMFA) (remfa.webnode.co.uk), which started in September 2020 and is ongoing. For the present analysis, we used records reported in the first 24 months (September 2020–2022) of REMFA's activity. REMFA is an initiative that, in addition to capturing roadkill data, seeks to promote citizen environmental education on road ecology. Using word of mouth as well as traditional and social media, we invited people to share photos and geographical locations of roadkills in Ecuador. Communications included emails, social networks [Facebook (https://www.facebook.com/profile.php?id=100063511063333), Instagram (https://instagram.com/remfa_fauna_silv_atrop?igshid=YmMyMTA2M2Y=) and Twitter (https://twitter.com/ratropellada)], messaging platforms (WhatsApp), and the mobile App Epicollect5 (the dedicated project is under the name “Animales atropellados Ecuador,” https://five.epicollect.net/project/animales‐atropellados‐ecuador). We also received data from a government entity, the Ministerio del Ambiente, Agua y Transición Ecológica del Ecuador. They provided roadkill data that is collected through their iNaturalist project (https://www.inaturalist.org/projects/atropellamiento‐de‐fauna‐silvestre‐de‐ecuador; License creative Commons Atribución – No Comercial – No Derivativa 4.0 CC BY‐NC‐ND 4.0). The main contributors to this project are Ministry personnel. Rangers from Parque Nacional Cayambe Coca zona baja also provided a previously compiled database of nonsystematic roadkill observations. Additionally, we included sporadic roadkill records found in the scientific literature but that had not been gathered via systematic surveys.

As several records are reported by nonbiologist citizens, misidentifications, or the use of common names are usual. Therefore, the REMFA team (biologists, taxonomists, and database manager) curated all records individually, confirming, correcting, or determining species identification (analyzing photographs provided with each record). When photographs or the deteriorated state of carcasses did not allow species identification, we assigned less specific taxonomic identification (e.g., genus, family, order, class). We checked for consistency of geographical data provided by users using Geographical Information Systems and known distribution areas of species consulting specialized literature (e.g., Ridgely & Greenfield, 2006; Tirira, 2017; Valencia et al., 2016) and IUCN and BIOWEB web platforms (IUCN, 2022; Pontificia Universidad Católica del Ecuador, 2021). Inconsistent data were removed.

In contrast to systematic monitoring, nonsystematic monitoring lacks standardized methodologies, and sampling effort is largely unreported, therefore estimating roadkill rates was not possible. Accounting for these limitations, we summarize data from nonsystematic studies by reporting the total number of records for identified taxonomic classes and species (when identified), and totals for each Ecuadorian province.

For systematic and nonsystematic data, when geographical coordinates of roadkill events were not available, we defined coordinates as the central point of the geographical reference provided by citizens or the published source. For these cases, we included an uncertainty value to account for the potential error in the estimated coordinates based on the described area or road. This was given in km when we had information on the location of the road where the roadkill was found, or in km^2^ when the road was not described, but we had information on the administrative area.

## 3 RESULTS

We compiled a total of 5010 roadkill events from both systematic and nonsystematic surveys (citizen science and other studies). Most of these records (85%, *n* = 4244) had accurate geographical information, with GPS coordinates taken at the site where the roadkill was found. In total, 3928 roadkill records had a species‐level identification and represented 392 wildlife species. Additionally, 1082 individuals were not identified at a species level due to the deteriorated state of carcasses or taxonomic uncertainties. For example, *Atractus* snakes and caecilians, which are poorly‐studied groups with large taxonomic uncertainties (Cisneros‐Heredia, 2005; Wilkinson, 2012), represented 88 and 74 unidentified individuals, respectively. Most identified species were nonthreatened fauna, but two are listed as Critically Endangered, four as Endangered, nine as Vulnerable, and six as Data Deficient by the IUCN Red List (IUCN, 2022). Nearly all records were tetrapods with relatively similar proportions in the four classes but marked differences in the diversity of identified species. With 1428 records (28.50% of the total), birds had the highest number of roadkills and included 181 species. Second were reptiles with 1356 records (27.06% of the total) from 106 species. Mammals corresponded to 1326 records (26.47% of the total) from 77 species. Amphibians were last, with 895 records (17.86%) from 28 species. Anecdotally, we obtained five records via citizen science (0.1%) for two invertebrate classes, Malacostraca and Clitellata. This vertebrate bias does not reflect a lack of roadkill among invertebrates but differences in detectability and a frequent focus on vertebrates as the target group in systematic and citizen science surveys.

Although amphibians had the lowest number of records, the cane toad *Rhinella marina* was the most roadkilled species with 532 records (59.44% of records for amphibians). The second and third most recorded species were two marsupials: the common opossum *Didelphis marsupialis* (*n* = 454, 34.23% of records for mammals) and the Andean white‐eared opossum *Didelphis pernigra* (*n* = 336, 25.34% of records for mammals) for a proportional subtotal of 64% within mammals. A bird, the yellow warbler *Setophaga petechia* (*n* = 193, 13.52% of records for birds), and a reptile, the common green iguana *Iguana iguana* (*n* = 126, 9.5% of records for reptiles), were the fourth and fifth most roadkilled species, respectively.

### 3.1 Systematic studies

We compiled 3305 wildlife roadkill records from 10 systematic surveys conducted on Ecuadorian roads. These corresponded to five published papers, four theses, and one unpublished dataset (Medrano‐Vizcaíno, Brito‐Zapata, & González‐Suárez, 2022). Georeferenced data were available for eight of these studies and comprised 2744 roadkill records.

**FIGURE 1:**
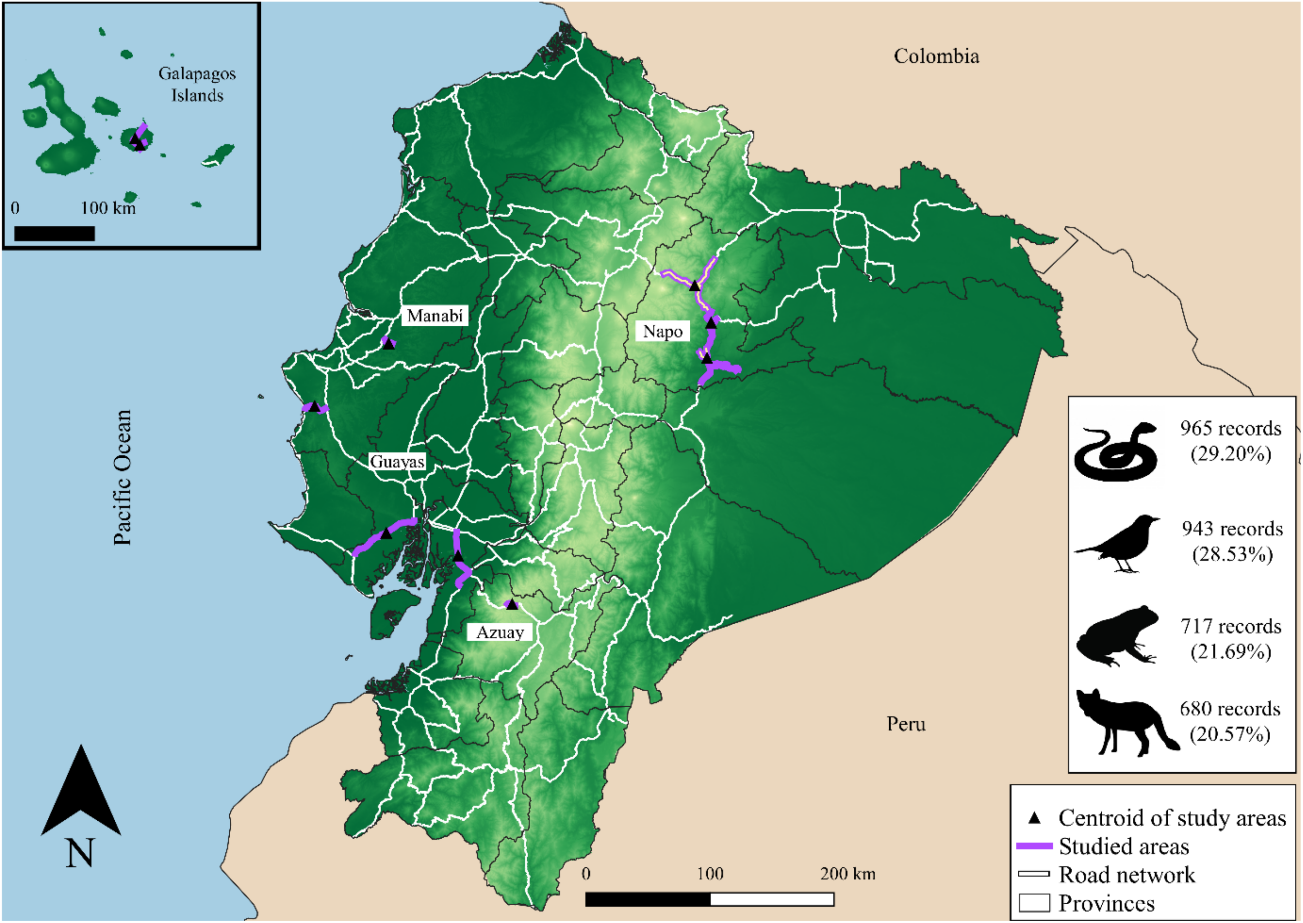
Ecuadorian road network (primary and secondary roads) highlighting the sites and roads where systematic studies were conducted and the total number of roadkill records per taxonomic class across all studies. For the Napo province, the study area of Medrano‐Vizcaíno, Brito‐Zapata, and González‐Suárez (2022) covers most of this province (thick purple line), overlapping with the study areas by Filius et al. (2020) and Medrano‐Vizcaíno and Espinosa (2021), both shown with a white line.

Systematic survey studies were not only rare but also geographically biased (Figure 1), with three studies conducted in the Napo province (Filius et al., 2020; Medrano‐Vizcaíno, Brito‐Zapata, & González‐Suárez, 2022; Medrano‐Vizcaíno & Espinosa, 2021), two in Galapagos (García‐Carrasco et al., 2020; Tanner & Perry, 2007), two in Guayas (Armendáriz, 2022; González, 2018), two in Manabi (Gaón & Valdez, 2021; Zavala, 2020), and one in Azuay (Aguilar et al., 2019).

Collectively systematic studies surveyed only 2.7% (454.5 km) of the 16,647.65 km of primary and secondary roads in Ecuador (Meijer et al., 2018) and yet reported roadkill events for at least 242 species (i.e., not all individuals were identified). Seven out of the 10 studies reported data for all tetrapods, while two studies focused on birds, and one on a single species: the lava lizard *Microlophus albemarlensis*. Most records were for reptiles (744 from 65 species; 221 unidentified individuals) and birds (674 from 117 species; 269 unidentified individuals), followed by amphibians (563 from 18 identified species; 154 unidentified individuals), and mammals (563 from 42 species; 117 unidentified individuals). Roads were monitored using different methods, which included driving a car, bicycle, motorcycle, and walking. Survey intervals varied between 1.4 and 7 days with the total survey period ranging from 27 to 425 days (Table 1).

**TABLE 1:** Systematic roadkill surveys conducted in Ecuador.

Province	Study	Studied taxa	Roadkills (*n*)	Road length (km)	Survey period (days)	Survey interval (days)	Survey method
Azuay	Aguilar et al. (2019)	Birds	60	15	365	7	Walking
Galapagos	Tanner and Perry (2007)	*Microlophus albemarlensis*	71	40	27	7	Motorcycle
Galapagos	García‐Carrasco et al. (2020)	Birds	168	13.8	30	2	Bicycle
Guayas	González (2018)	Tetrapods	224	51	425	3.5	Car
Guayas	Armendáriz (2022)	Tetrapods	246	58.1	273	7	Car
Manabi	Zavala (2020)	Tetrapods	52	26.6	119	3.5	Car
Manabi	Gaón & Valdez (2021)	Tetrapods	321	10	91	1.4	Walking
Napo	Filius et al. (2020)	Tetrapods	590	15.88	121	1.4	Bicycle
Napo	Medrano‐Vizcaíno and Espinosa (2021)	Tetrapods	445	99	168	2.3	Car
Napo	Medrano‐Vizcaíno, Brito‐Zapata, and González‐Suárez (2022)	Tetrapods	1125	240	185	1.4	Car

*Note*: For each study, we list the province where data were collected, citation, studied taxonomic group, total number of roadkill records, length of road surveyed, survey period, survey interval, and survey method.

Estimates of the 333 standardized corrected roadkill rates varied greatly across the 242 analyzed species (median = 0.45 ind./km/year, SD = 11.92 ind./km/year), with several taxa, particularly birds and reptiles, estimated to suffer high mortality rates in Galapagos and Napo (Table 2; Appendix 1). Although most roadkilled species were categorized as Least Concern by the IUCN Red List (IUCN, 2022), six species were found in a category of conservation concern, and two as Data Deficient (Table 3). While standardized corrected roadkill rates for these threatened species were not high, due to their vulnerability, road mortality could pose a threat to their persistence. Although the tapeti (*Sylvilagus brasiliensis*), considered as Endangered, was reported in four studies with a median roadkill rate of 0.028 ind./km/year (range = 0.008–0.79), it is likely that these records represent other *Sylvilagus* species, as the IUCN restricts the distribution of *S. brasiliensis* to Pernambuco‐Brazil (IUCN, 2022).

**TABLE 2:** Top 10 most roadkilled species in Ecuador as reported in systematic surveys.

Class	Order	Species	IUCN status	No. of studies	Roadkill rate (ind./km/y)	Province
Aves	Passeriformes	*Setophaga petechia*	LC	1	171.72	Galapagos
Amphibia	Anura	*Rhinella marina*	LC	5	110.70	Manabi
Reptilia	Squamata	*Microlophus albemarlensis*	LC	1	47.17	Galapagos
Aves	Passeriformes	*Geospiza fuliginosa*	LC	1	24.53	Galapagos
Mammalia	Didelphimorphia	*Didelphis pernigra*	LC	2	23.67	Napo
Reptilia	Squamata	*Iguana iguana*	LC	4	18.75	Guayas
Reptilia	Squamata	*Amphisbaena fuliginosa*	LC	2	18.72	Napo
Reptilia	Squamata	*Atractus collaris*	LC	1	13.89	Napo
Mammalia	Didelphimorphia	*Didelphis marsupialis*	LC	5	13.88	Manabi
Aves	Cuculiformes	*Crotophaga ani*	LC	6	12.27	Galapagos

*Note*: We present taxonomic information (class, order, and species), IUCN Red List status, number of studies in which the species was recorded, estimated standardized corrected roadkill rate (if a species was detected in more than one study, we report its highest rate), and the province for the reported rate.

**TABLE 3:** Species listed as threatened or data deficient by the IUCN Red List or not yet unassessed, which were reported as roadkill by systematic surveys in Ecuador.

Class	Order	Species	IUCN status	No. of studies	Roadkill rate (ind./km/y)	Province
Mammalia	Lagomorpha	*Sylvilagus brasiliensis*	EN	3	1.19	Manabi
Aves	Apodiformes	*Metallura baroni*	EN	1	0.26	Azuay
Reptilia	Squamata	*Coniophanes dromiciformis*	VU	1	1.91	Manabi
Mammalia	Carnivora	*Leopardus tigrinus*	VU	1	0.14	Napo
Mammalia	Squamata	*Trilepida anthracina*	VU	1	0.08	Napo
Mammalia	Cingulata	*Priodontes maximus*	VU	1	0.05	Napo
Mammalia	Rodentia	*Ichthyomys tweedii*	DD	1	5.11	Manabi
Reptilia	Squamata	*Atractus touzeti*	DD	1	0.17	Napo
Mammalia	Lagomorpha	*Sylvilagus andinus*	DD	1	0.06	Napo
Reptilia	Squamata	*Dipsas georgejetti*	NE	1	0.29	Guayas

*Note*: We provide taxonomic information (class, order, and species name), IUCN Red List status, the number of studies in which that species was recorded, the estimated corrected roadkill rate (if a species was detected in more than one study, we report its highest corrected rate), and the province for the reported rate.

### 3.2 Citizen science and other records

**FIGURE 2:**
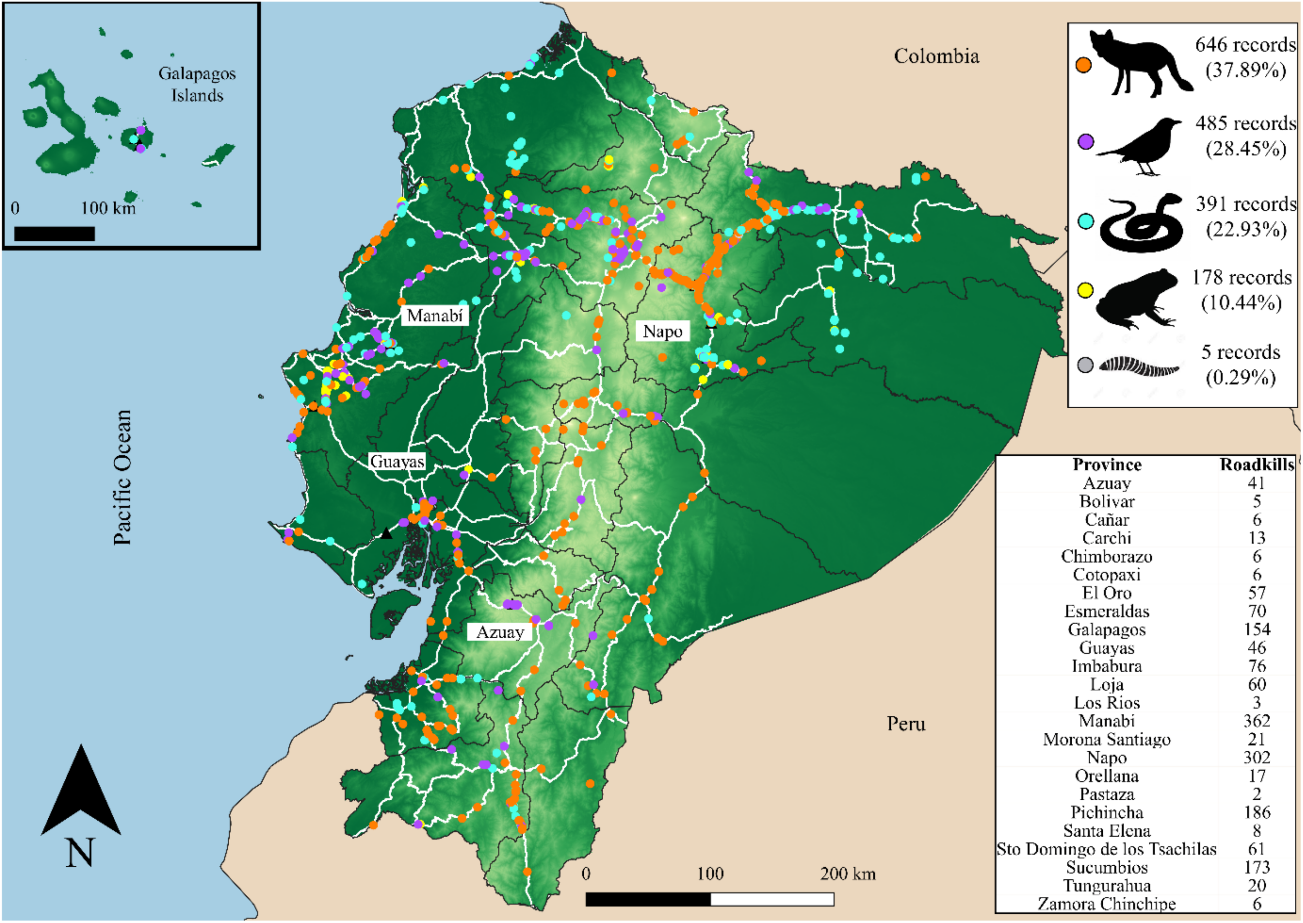
Location of all roadkill records compiled from citizen science and other sources and total number of records for each taxonomic Class and province

Citizen science and other nonsystematic records provided a smaller sample of 1705 roadkill records but offered a much wider geographical coverage than the systematic data with records from all 24 provinces of Ecuador (Figure 2). Most records were from Manabi province (362), Napo (302), and Pichincha (186). Records were mainly obtained from the REMFA citizen science project (698 records), iNaturalist (556 records), and from seven scientific studies that reported 154 roadkill records collected sporadically, not in a systematic survey. Our initiative REMFA offers different ways to report roadkill, and we found a difference in frequency of use, with most data reported via WhatsApp (457 records), followed by the Epicollect App (165 records), Social networks (Facebook, Twitter, and Instagram; 73 records), and Email (3 records).

Among all nonsystematic records, mammals were the most reported group (646 records, 53 identified species; 80 unidentified individuals), followed by birds (485 records, 116 species; 76 unidentified individuals), reptiles (391 records, 76 species; 93 unidentified individuals) and amphibians (178 records, 17 species; 67 unidentified individuals). Two marsupial mammals (*D. marsupialis and D. pernigra*) and the yellow warbler (*S. petechia*) were the most frequently reported species (250, 104, and 81, respectively. Table 4). Several species were reported from multiple locations and provinces, with *D. marsupialis* roadkill reports from 16 provinces, *D. pernigra* from 11, *Tamandua mexicana* and *Conepatus semistriatus* from nine, and *Coragyps atratus* from eight provinces.

**TABLE 4:** Top 10 roadkilled wildlife in Ecuador based on citizen science and other sources (nonsystematic studies).

Class	Order	Species	IUCN status	No. of roadkills
Mammalia	Didelphimorphia	*Didelphis marsupialis*	LC	250
Mammalia	Didelphimorphia	*Didelphis pernigra*	LC	104
Aves	Passeriformes	*Setophaga petechia*	LC	81
Amphibia	Anura	*Rhinella marina*	LC	53
Reptilia	Squamata	*Atractus dunni*	NT	37
Aves	Cathartiformes	*Coragyps atratus*	LC	37
Mammalia	Carnivora	*Conepatus semistriatus*	LC	25
Reptilia	Squamata	*Iguana iguana*	LC	23
Reptilia	Squamata	*Boa imperator*	LC	23
Reptilia	Squamata	*Epicrates cenchria*	LC	20

*Note*: We report taxonomic information (class, order, and species names), IUCN Red List status, and the total number of roadkill records.

As with the systematic data, most records represented species classified as Least Concern by the IUCN Red List (IUCN, 2022), but there were 12 species of conservation concern, four currently listed as Data Deficient, and one not yet assessed by the IUCN (Table 5).

**TABLE 5:** Species listed as threatened or data deficient by the IUCN Red List or not yet unassessed, which were reported as roadkill by citizen scientists and in nonsystematic studies in Ecuador.

Species	IUCN status	No. of roadkills
*Chelonoidis porteri*	CR	1
*Pterodroma phaeopygia*	CR	1
*Porthidium arcosae*	EN	3
*Metallura baroni*	EN	1
*Tapirus pinchaque*	EN	1
*Ceratophrys stolzmanni*	VU	7
*Coniophanes dromiciformis*	VU	4
*Dipsas elegans*	VU	2
*Leopardus tigrinus*	VU	2
*Mazama rufina*	VU	2
*Alouatta palliata*	VU	2
*Chelonoidis denticulata*	VU	1
*Sylvilagus andinus*	DD	7
*Chironius flavopictus*	DD	4
*Coendou quichua*	DD	2
*Synophis calamitus*	DD	1
*Dipsas georgejetti*	NE	13

*Note*: We provide species name, IUCN Red List status, and the number of roadkill records.

## 4 DISCUSSION

We compiled a large dataset that describes mortality due to wildlife‐vehicle collision in Ecuador based on both systematic surveys and nonsystematic records that came from citizen science and opportunistic observations reported in the scientific literature. Collectively, these data reveal that 11.79% of described vertebrate species from Ecuador are susceptible to die on roads, a number that likely underestimates the true impact of roads as not all individuals could be identified at the species level, not all areas are well sampled, and smaller and cryptic species may be underreported. We see a need for additional systematic surveys, which can provide more comparable estimates but so far have been few and limited to some areas. Likewise, citizen science reports are overrepresenting certain areas, with gaps in other regions.

We found that marsupials (*D. marsupialis* and *D. pernigra*) represented more than half of mammalian records (systematic and nonsystematic) reported in 21 out of the 24 provinces. Their generalist habits (diet and habitat), together with increased abundance and intermediate body masses could be important factors for their high mortality (Medrano‐Vizcaíno, Grilo, et al., 2022). Likewise, species with a scavenger or omnivorous diet such as the American black vulture *Coragyps atratus*, the smooth‐billed ani *Crotophaga ani*, and the groove‐billed ani *Crotophaga sulcirostris* comprised a great part of the avian records. The common green iguana *Iguana iguana* was the most common roadkilled reptile. While this is mainly a herbivore, iguanas have been recorded feeding on carcasses of deer and opossums (Anderson & Enge, 2012). Roadkilled animals and invertebrates can be a food source for scavengers that are attracted to roads increasing their risk of being hit by a car.

Among the systematic studies, we estimated particularly high standardized roadkill rates in some cases. Studies in Galapagos (García‐Carrasco et al., 2020), Napo (Filius et al., 2020), and Manabi (Gaón & Valdez, 2021) reported roadkill rates that were disproportionally high in comparison with other studies and were all conducted in small areas (13.8, 15.8, and 10 km, respectively). These areas could be hotspots of mortality that do not represent rates across wider areas (overestimate risk), nevertheless, how road impacts wildlife in areas of biological importance should be further studied.

For example, high road mortality for endemics of the Galapagos Islands could threaten population persistence (Tejera et al., 2018; Wiedenfeld, 2006). In this territory, threatened species comprise 20 out of 43 bird species, 18 out of 42 reptile species, and 6 out of 9 noncetacean mammals species (IUCN, 2022), for which the impacts of roads on their populations remain unexplored but also nonthreatened species may be impacted. For example, the yellow warbler, *Setophaga petechia*, that was commonly reported as roadkill, has experienced a dramatic population decline, and although low insect abundance due to intense use of herbicides has been associated with this decline (Dvorak et al., 2012), road mortality could be a significant factor contributing to the observed decline. While there have been two systematic studies in Galapagos, these offer limited insight, as one was focused on a single species (Tanner & Perry, 2007) and the other represented a single one‐month survey (García‐Carrasco et al., 2020). The real impact of roads on wildlife populations in Galapagos remains unknown. Considering the high endemism and quantity of threatened species, we think the roads in the Galapagos islands deserve special attention for research.

We found roadkill records for 15 Threatened and six Data Deficient species, with some of them showing repeatedly in different regions of Ecuador. The Peters' running snake *Coniophanes dromiciformis*, is a Vulnerable and poorly‐studied species, known from only nine locations of Ecuador (Cisneros‐Heredia, 2021). This range‐restricted species showed a roadkill rate of 1.91 ind./km/year in the province of Manabi and was reported four times in our nonsystematic records. Another worrying case is the Violet‐throated Metaltail *Metallura baroni*. This bird, catalogued as Endangered and endemic to the Azuay and Cañar provinces, is known from only five locations (BirdLife International, 2016). We estimated a roadkill rate for this species of 0.26 ind./km/year in Azuay. In addition, we gathered seven nonsystematic records of the pacific horned frog *Ceratophrys stolzmanni* in Manabi, a Vulnerable and rare species whose entire population is distributed in less than eight subpopulations (IUCN SSC Amphibian Specialist Group, 2018). Directing research efforts on roads within the distribution areas of these range‐restricted species could be important to determine the impact of roads on their populations.

Additionally, poorly‐studied organisms such as Caecilians and *Atractus* sp. snakes were also regularly reported as roadkill. These animals are among the least known vertebrates, with several taxonomic uncertainties and unknown conservation status for many of them (Cisneros‐Heredia, 2005; IUCN, 2022; Wilkinson, 2012). Both groups were frequently detected across three studies in Napo (Filius et al., 2020; Medrano‐Vizcaíno, Brito‐Zapata, & González‐Suárez, 2022; Medrano‐Vizcaíno & Espinosa, 2021), and nonsystematic data included 41 records of *Atractus* sp. snakes. In a survey conducted in 2014, Medrano‐Vizcaíno and Espinosa (2021) found two roadkilled individuals attributed to the genus *Atractus* in Napo, which were latter described as new species (described by Melo‐Sampaio et al., 2021; Arteaga et al., 2022). Road ecology research in areas with poorly known and undescribed species would be valuable, both to quantify impacts but also to strengthen our understanding of these species.

Our study offers an overview of wildlife mortality on Ecuadorian roads but likely underestimates the impact of roads on species. We obtained roadkill records from all 24 provinces in Ecuador, but systematic studies were only available for five, and nonsystematic data were disproportionally distributed in provinces like Manabi, and Napo (more than 300 records for each province). While in the provinces of Bolivar, Cañar, Carchi, Cotopaxi, Galapagos, Los Rios, Pastaza, Santa Elena, and Zamora Chinchipe, we gathered fewer than 10 records per province. Our results also show that citizen science reports can corroborate results from systematic studies to identify top roadkill species. *D. marsupialis, D. pernigra, S. petechia*, and *R. marina* were both commonly reported in nonsystematic and systematic data from Galapagos, Manabi, Guayas, and Napo. We hope that by expanding our network of citizen scientists in REMFA, we will be able to fill geographical gaps of information and gain insights into wildlife mortality in areas where systematic studies are lacking.

A limitation in our compiled dataset is the assumption of correct taxonomic identification in systematic studies (and our own correct identification in nonsystematic records). For example, some species were reported outside their known distribution ranges in Ecuador: *Neacomys amoneus, Mesoclemmys heliostemma, Myrmochanes hemileucus, Pseudocolopteryx acutipennis, Rhogeessa io, Scinax ruber, and Xenoxybelis argenteus*, and even one species that is assumed to be not distributed in Ecuador was reported (*Rhinella arenarum*). These may be misidentifications (likely for *R. arenarum*), but previous roadkilled specimens have revealed new distribution areas for certain species (Medrano‐Vizcaíno & Brito‐Zapata, 2021). Roadkill records can provide valuable information about the biology and ecology of species, and with correct taxonomic identification, can contribute to our understanding of biodiversity.

Road ecology research in Ecuador is gaining interest, but it is still relatively limited. Promoting and guiding additional research and public engagement is important. Through our citizen science project REMFA we have given a special relevance to science communication, which has been vital to reach citizens to join our work, and we are now engaging with policymakers. The active involvement of government institutions such as the Ministry of Environment, Water and Ecological Transition, together with the Ministry of Public Works is necessary for the inclusion of adequate policies to reduce wildlife mortality across existing roads, and to plan sustainable roads for the future. We hope that this work can be an initial step towards these national aims for wildlife conservation.

## AUTHOR CONTRIBUTIONS

**Pablo Medrano‐Vizcaíno:** Conceptualization (lead); data curation (lead); formal analysis (lead); funding acquisition (lead); investigation (lead); methodology (lead); project administration (lead); resources (lead); validation (lead); visualization (lead); writing – original draft (lead); writing – review and editing (lead). **David Brito‐Zapata:** Conceptualization (supporting); data curation (equal); project administration (supporting); writing – review and editing (supporting). **Adriana Rueda:** Data curation (equal); visualization (equal). **Pablo Jarrín‐V:** Resources (equal); writing – review and editing (supporting). **José‐María García‐Carrasco:** Resources (supporting); visualization (supporting); writing – review and editing (supporting). **Diana Medina:** Resources (supporting); visualization (supporting). **Juan Aguilar:** Resources (supporting). **Néstor Acosta‐Buenaño:** Resources (supporting). **Manuela González‐Suárez:** Conceptualization (supporting); methodology (supporting); supervision (lead); visualization (equal); writing – review and editing (equal).

## CONFLICT OF INTEREST STATEMENT

The authors declare no conflicts of interest.

### OPEN RESEARCH BADGES

This article has earned an Open Data badge for making publicly available the digitally‐shareable data necessary to reproduce the reported results. The data is available at https://doi.org/10.6084/m9.figshare.21313650.

## Data Availability

We provide 333 raw and corrected roadkill rates calculated for 242 species found in systematic studies as Appendix 1 and a database of 5010 roadkill records (4244 with accurate geographical coordinates taken at the roadkill site) from 392 species as Appendix 2. Full dataset is freely available for download at: https://doi.org/10.6084/m9.figshare.21313650. This public database will be quarterly updated at REMFA's webpage: https://remfa.webnode.co.uk/, where a link for download will be posted.
